# Thoraco-abdominal coordination and performance during uphill running at altitude

**DOI:** 10.1371/journal.pone.0174927

**Published:** 2017-03-31

**Authors:** Eva Bernardi, Lorenza Pratali, Gaia Mandolesi, Maria Spiridonova, Giulio Sergio Roi, Annalisa Cogo

**Affiliations:** 1 Biomedical Sport Studies Centre, University of Ferrara, Ferrara, Italy; 2 Institute of Clinical Physiology, National Research Council, Pisa, Italy; 3 Isokinetic Medical Group, Education and Research Department, Bologna, Italy; University of France Comté, FRANCE

## Abstract

**Introduction:**

Running races on mountain trails at moderate-high altitude with large elevation changes throughout has become increasingly popular. During exercise at altitude, ventilatory demands increase due to the combined effects of exercise and hypoxia.

**Aim:**

To investigate the relationships between thoraco-abdominal coordination, ventilatory pattern, oxygen saturation (SpO_2_), and endurance performance in runners during high-intensity uphill exercise.

**Methods:**

Fifteen participants (13 males, mean age 42±9 yrs) ran a “Vertical Kilometer,” i.e., an uphill run involving a climb of approximately 1000 m with a slope greater than 30%. The athletes were equipped with a portable respiratory inductive plethysmography system, a finger pulse oximeter and a global positioning unit (GPS). The ventilatory pattern (ventilation (VE), tidal volume (VT), respiratory rate (RR), and VE/VT ratio), thoraco-abdominal coordination, which is represented by the phase angle (PhA), and SpO_2_ were evaluated at rest and during the run. Before and after the run, we assessed respiratory function, respiratory muscle strength and the occurrence of interstitial pulmonary edema by thoracic ultrasound.

**Results:**

Two subjects were excluded from the respiratory inductive plethysmography analysis due to motion artifacts. A quadratic relationship between the slope and the PhA was observed (r = 0.995, p = 0.036). When the slope increased above 30%, the PhA increased, indicating a reduction in thoraco-abdominal coordination. The reduced thoraco-abdominal coordination was significantly related to reduced breathing efficiency (i.e., an increased VE/VT ratio; r = 0.961, p = 0.038) and SpO_2_ (r = -0.697, p<0.001). Lower SpO_2_ values were associated with lower speeds at 20%≥slope≤40% (r = 0.335, p<0.001 for horizontal and r = 0.36, p<0.001 for vertical). The reduced thoraco-abdominal coordination and consequent reduction in SpO_2_ were associated with interstitial pulmonary edema.

**Conclusion:**

Reductions in thoraco-abdominal coordination are associated with a less efficient ventilatory pattern and lower SpO_2_ during uphill running. This fact could have a negative effect on performance.

## Introduction

Running races on mountain trails at moderate-high altitude with large elevation changes throughout has become increasingly popular. High-intensity exercise under these environmental conditions requires extensive respiratory and cardiovascular system engagement. The respiratory and cardiac responses that occur during this type of exercise represent key constituents of endurance performance that can be affected by oxygen desaturation and increased respiratory work [1–3]. Indeed, during high-intensity exercise at altitude, ventilatory demands increase due to the combined effects of exercise and hypoxia, which affect the ventilatory system above 2000–2500 m [4–6]. Under such conditions, the respiratory muscles become strained and can experience progressive fatigue, and lung function can be impaired [3,7]. The postural tasks related to the presence of steep slopes and different types of terrain [8] may also affect the ventilatory system through modifications of thoraco-abdominal coordination, which involves the coordinated action of the diaphragm and the abdominal muscles to inflate and deflate the chest. A reduction in thoraco-abdominal coordination may in turn alter ventilatory patterns and reduce ventilatory efficiency [9]. Ventilatory efficiency is defined as the amount of ventilation (VE) required to achieve a given level of oxygen saturation (SpO_2_) during spontaneous breathing [10]. Specifically, ventilatory patterns characterized by a higher tidal volume (VT) and reduced respiratory rate (RR) have been found to be the most efficient due to the reduction in dead space ventilation [11–14]. This ventilatory pattern can play an important role in SpO_2_ and therefore can influence respiratory and cardiac responses and exercise performance at altitude [10,12].

Ventilatory pattern and thoraco-abdominal coordination are important for determining ventilatory efficiency and can represent a key tool for functional evaluations of athletes’ performance. To the best of our knowledge, despite the importance of these aspects, few studies have examined ventilatory pattern and thoraco-abdominal motion in healthy adults both at rest and during exercise [15–17]. Romei et al. [16] showed that chest wall kinematics and ventilatory pattern are significantly influenced by body position during rest in healthy subjects. However, no study has focused on changes in these parameters during dynamic changes in body position.

Moreover, the development of interstitial pulmonary edema due to both strenuous exercise and hypoxia [18,19] can contribute to changes in lung function. Studies have frequently shown a significant increase in pulmonary extravascular water following strenuous exercise, leading to mild interstitial pulmonary edema. Although small increases in interstitial water in the lungs do not cause overt clinical symptoms, they could contribute to lung-related limitations to exercise performance [20]. Interstitial pulmonary edema has been shown in endurance athletes after intense exercise at sea level using different imaging techniques such as chest X-ray, magnetic resonance imaging, computed tomography, and scintigraphy [21–23]. Currently, chest ultrasounds are a common method to detect the presence of pulmonary extravascular water based on the presence of multiple diffuse bilateral B lines [24]. Ultrasound B lines (US-B lines) are considered a sonographic sign of lung interstitial syndrome. US-B lines are defined as discrete laser-like vertical hyperechoic reverberation artifacts that arise from the pleural line, extend to the bottom of the screen without fading, and move synchronously with the sliding of the lung [24].

In the present study, we hypothesized that during strenuous uphill exercise at moderate altitude, the preservation of a coordinated movement between the chest wall and abdomen (i.e., the two compartments move synchronously during the ventilatory cycle) would be associated with more efficient breathing, higher SpO_2_ levels and reduced ventilatory demands. Moreover, we hypothesized that all these factors would influence endurance performance and cardiorespiratory responses. We predicted that steeper slopes might prompt subjects to change their postures, leading to reductions in thoraco-abdominal coordination and the above-reported effects.

To examine these hypotheses, we tested competitive athletes taking part in skyrunning during an uphill run above 2000 m. Skyrunning involves running on mountain trails at an altitude of at least 2000 m and with ground slopes that can exceed 30%. Respiratory function and the effects of increased slopes on thoraco-abdominal coordination and breathing efficiency were examined via analyses of ventilatory parameters and SpO_2_, which was measured via finger pulse oximetry (a method widely utilized and validated for field research) [25]. We also assessed the possible occurrence of interstitial pulmonary edema via thoracic ultrasound as a contributing factor limiting performance under these conditions.

## Materials and methods

Fifteen endurance-trained, nationally competitive skyrunners (13 males) were recruited with the aid of the International Skyrunning Federation. The study was conducted in accordance with the Declaration of Helsinki and was approved by the Ethics and Research Committee of the Medical School of the University of Ferrara (protocol number 120387). All participants provided their informed written consent to participate in the study. The exclusion criteria were injuries and illnesses that impaired normal training in the three months prior to the study. No participant was excluded.

### Experimental overview

The athletes performed a simulated individual Vertical Kilometer, i.e., a run with approximately 1000 m of vertical climbing over variable terrain with a substantial incline, not exceeding five kilometers in length (International Skyrunning Federation rule 2.4.8) and with slopes that could exceed 30%. The simulated Vertical Kilometer was shortened to 794 m (from Cervinia, Italy, at 2030 m to a place slightly above Rifugio Oriondè, Italy, at 2824 m) due to the presence of snow on the upper part of the trail. Along the route, the slopes varied from 2% up to 40% [26] with variable changes along the way. In fact, steep slopes were situated at the beginning as well as at the end of the trail (a detailed description is available in the website of Vertical Kilometer, www.cervinoxtrail.com). Immediately after the arrival at Rifugio Oriondè, the athletes ran back downhill to the starting point.

Anthropometric data, medical histories and altitude training information were collected using a questionnaire.

### Respiratory function

Vital capacity (VC), forced expiratory volume in the first second (FEV_1_), FEV_1_/VC ratio and inspiratory muscle strength (PImax) were measured at rest, before the uphill run (T0) and immediately after the descent (T1) using a portable spirometer (Spiropalm Cosmed; Roma, Italy) and a respiratory pressure meter (MicroRPM Carefusion, UK). All tests were performed according to the ATS/ERS guidelines [27,28]. The predicted reference values used were the values reported in the Global Lung Function Initiative [29].

### Ventilation monitoring

During the event, the athletes were outfitted with a portable respiratory inductive plethysmography system (Lifeshirt VivoMetrics, Inc, Ventura CA, USA), which consisted of a snugly fitting elastic garment that incorporated rib cage and abdominal inductance sensors containing shielded electrical conductors connected to signal-processing and recording unit for continuous data collection. A finger pulse oximeter was integrated in the system [30]. The sensors were placed by the same investigator for consistency. The recorded data included changes in ventilatory pattern (VE, RR, VT and VE/VT ratio) and thoraco-abdominal coordination, which was expressed as the phase angle index (PhA). The VE/VT ratio is useful for describing the ventilatory response to exercise and indicating the contribution of VT to VE during exercise [31, 32]. These parameters were measured breath-by-breath throughout the run. The PhA was assessed by computing the contribution of the rib cage to VT and the phase shift between the rib cage and the abdominal excursion. A value of 0° indicates total synchrony, and a value of 180° indicates total asynchrony between the two compartments. The system was calibrated for each subject at rest before the event. Following the calibration procedure, the subjects were asked to breathe rapidly into a calibration bag with a fixed volume for seven breathing cycles that involved emptying and filling the bag with each breath. This procedure was repeated four times while alternating between sitting and upright positions to evaluate the contributions of the rib cage and abdomen to the VT at different body positions.

### GPS

A global positioning system unit (GPS; Garmin Edge 305, USA) was positioned on the arm of the subjects to record the path traveled, altitude, slope and horizontal speed. The Garmin Edge 305 is a satellite-based navigation system provided with an antenna that acquires satellite information and a barometric altimeter to calculate the elevation. The system records the point-to-point distance; the horizontal speed is calculated as the ratio of distance over time and the slope as the change in elevation over the course of the run. The vertical speed can be subsequently calculated as the ratio of difference in height over time. The reported accuracy of this GPS model is 5 m for both speed and distance. GPS errors can arise in obstructed conditions, such as indoors or in a narrow valley, but these scenarios were not the case for the run from Cervinia to Rifugio Oriondè. Other potential sources of GPS inaccuracy are atmospheric disturbances, but the present study was performed under good weather conditions and with no drop in the barometric pressure.

### Ultrasound lung echo

To diagnose the presence of pulmonary extravascular water, ultrasound lung echo assessments were performed. This technique has been compared to gold standard methods [33–35], and many studies have addressed its use in the diagnosis of pulmonary edema, particularly in non-hospital settings [36–39]. Ultrasound chest examinations (Vivid I, General Electric Healthcare Clinical System, Buckinghamshire, UK) were performed by an experienced cardiologist (L.P.) 30 min before the run and within 10 min after the descent to assess for the presence of interstitial pulmonary edema, which was identified based on US-B lines using a cardiac probe (2.5–3.5 MHz). The cardiologist was unaware of the results of the pulmonary function test, SpO_2_ or the subjects’ performance. A total of 28 echographic windows were scanned in each anterior and lateral hemithorax from the second to the fourth (on the right side to the fifth) intercostal spaces and from the parasternal line to the axillary line. In each intercostal space, the number of US-B lines signs was recorded at the parasternal, midclavicular anterior axillary, and midaxillary sites. The sum of the US-B lines indicates the extent of extravascular fluid in the lungs. Zero is defined as a complete absence of US-B lines in the investigated area. A score of ≤ 5 US-B lines was defined as a normal echographic chest pattern [40].

### Data and statistical analysis

The VE, PhA and SpO_2_ data were analyzed with dedicated software (VivoLogic, VivoMetrics, Inc, Ventura CA, USA).

Only data regarding the ascent were analyzed. Movement artifacts introduced during exercise were removed from the collected data by setting the minimal acceptable VT values for breath assessment to 25% of the basal value (i.e., for a VT of 400 mL a deviation less than 100 mL was not considered a breath). Similarly, we removed low SpO_2_ values if they were not supported by a reasonable heart rate and were therefore likely due to poor signal or motion artifact. The ventilatory pattern (VE, VT and RR), PhA and SpO_2_ data were averaged over one-minute intervals and integrated with the GPS data. To simplify the graphic representation in the X-Y axis, we grouped all values into different classes. The slope values were grouped into four classes (i.e., 0–10%, 10–20%, 20–30%, and 30–40%). The same procedure was applied to the PhA values (i.e., 0–10, 10–13, 13–16, and >16) and the delta SpO_2_ values, which were expressed as the difference between the SpO_2_ values at rest and those during exercise (i.e., ≥-15, <-15 ≥-10, <-10 ≥-5 and <-5 ≥0). All computations were performed using a statistical software package (SPSS version 20.0.0; IBM Corp., Armonk, NY, USA).

Normal distributions of the data were verified using the Kolmogorov-Smirnov test. Polynomial regression was used to test the nonlinear relationships of the slope with the PhA. For all ventilatory parameters, mean values and 95% confidence intervals (CI) were calculated. The differences between the means of each parameter measured at T0 and T1 were evaluated using paired Student’s t tests. The effect size was calculated as the standardized mean difference in respiratory function, ventilatory pattern and SpO_2_ data between T0 and T1 and graded according to Cohen’s *d*. Correlations were analyzed based on Pearson correlation coefficients for the dependent variables. Multivariable logistic regression analysis was performed considering speed as the dependent variable and the decrease in SpO_2_, the increase in VE/VT ratio, the PhA and the slope as independent variables. The level of significance was set at p<0.05 for all statistical comparisons.

## Results

Fifteen experienced and trained skyrunners (13 males, mean age 42±9 yrs, mean body mass index 21.7±2.2 kg/m^2^) participated in the study. One male and one female were excluded from the respiratory inductive plethysmography analysis due to motion artifacts. The final study population included thirteen subjects (12 males) for the respiratory analysis while the spirometric and echo results refer to the whole group (15 subjects).

The athletes ran uphill for an average of 65.2±14.1 min at a mean horizontal speed of 5.7±1.1 km/h and a mean vertical speed of 792±158 m/h while simulating the Vertical Kilometer. A significant correlation was found between horizontal and vertical speed (p = 0.01). With respect to the downhill run, subjects spent a mean of 30±2.5 min descending to the starting point.

All subjects exhibited normal spirometric values (FEV_1_, VC, FEV_1_/VC ratio) and PImax at T0. VC (p = 0.041) and PImax (p = 0.023) were significantly reduced at T1 compared to T0, although the effect size was trivial (Table 1). The ventilatory pattern (VE, VT and RR) and SpO_2_ data at rest and during the run, expressed as the mean ± CI, are reported in Table 2. We observed a significant and high reduction in SpO_2_ during the run (p<0.001), in addition to the obvious increase in VE and the ventilatory pattern components.

**Table 1:** Respiratory function of 15 subjects before (T0) and after the run (T1).

	T0	T1	P	Effect size (d)
VC (L)	**5.5 ± 0.4**	**5.3 ± 0.4**	**0.041**	**0.14**
VC (% pred)	**117 ± 7**	**114 ± 8**	**0.041**	**-**
FEV_1_ (L)	4.1 ± 0.3	4.1 ± 0.2	0.921	0.0
FEV_1_ (% pred)	109 ± 4	110 ± 5	0.968	**-**
FEV_1_/VC	76.6 ± 9.0	75.3 ± 3.8	0.716	0.13
FEV_1_/VC (% pred)	93 ± 6	94 ± 5	0.744	**-**
PImax (cmH_2_O)	**115 ± 11**	**110 ± 11**	**0.023**	**0.22**

Data are expressed as the mean ± 95%CI.

Abbreviations: T0, at rest before the run; T1, at rest after the descent. VC, vital capacity; VC (% pred), % of predicted value of vital capacity; FEV_1_, forced expiratory volume in the first second; FEV_1_ (% pred), % of predicted value of forced expiratory volume in the first second; PImax, maximal inspiratory pressure.

**Table 2:** Ventilatory pattern and oxygen saturation of 13 subjects at rest (T0) and during the run.

	Rest	Race Run	P	Effect size (d)
VE (L/min)	**20.5 ± 2.5**	**131.1 ± 2.4**	**<0.001**	**0.94**
VT (L)	**0.9 ± 0.06**	**2.5 ± 0.03**	**<0.001**	**0.91**
RR (breaths/min)	**24.3 ± 2.7**	**52.8 ± 0.7**	**<0.001**	**0.89**
SpO_2_ (%)	**94.6 ± 0.6**	**85.2 ± 0.29**	**0.011**	**0.90**

Data are expressed as the mean ± 95%CI.

Abbreviations: VE, ventilation; VT, tidal volume; RR, respiratory rate; SpO_2_, oxygen saturation.

Only slopes between 0 and 40% were analyzed, as slopes >40% required climbing activity with the use of the hands, which involved unavoidable changes in posture.

**Fig 1:**
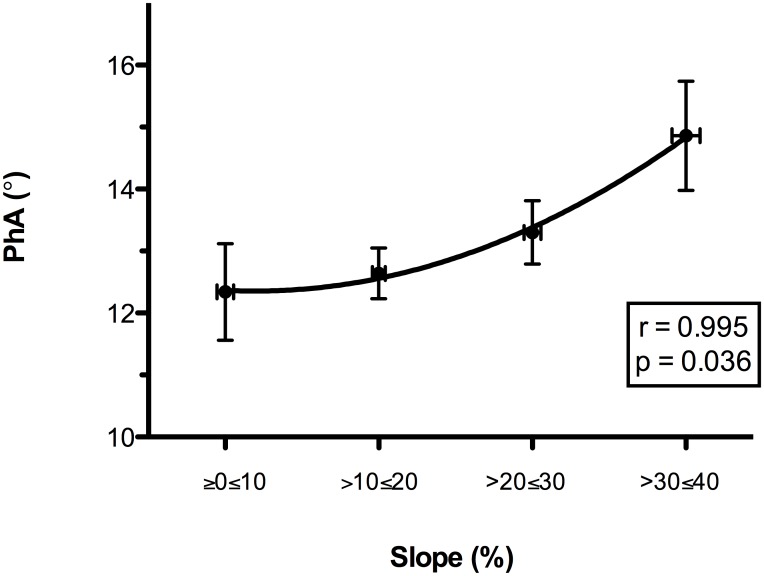
Relationship between ground slope and thoraco-abdominal coordination (PhA) during the run. The ground slope values were grouped into four classes, each associated with a mean PhA value. When the slope increased to over 30%, a significant increase in PhA was observed, representing a decrease in thoraco-abdominal coordination. The best interpolation of these points is represented by the quadratic curve PhA = 12.897–0.773slope + 0.301slope^2^. Abbreviations: PhA, phase angle.

**Fig 2:**
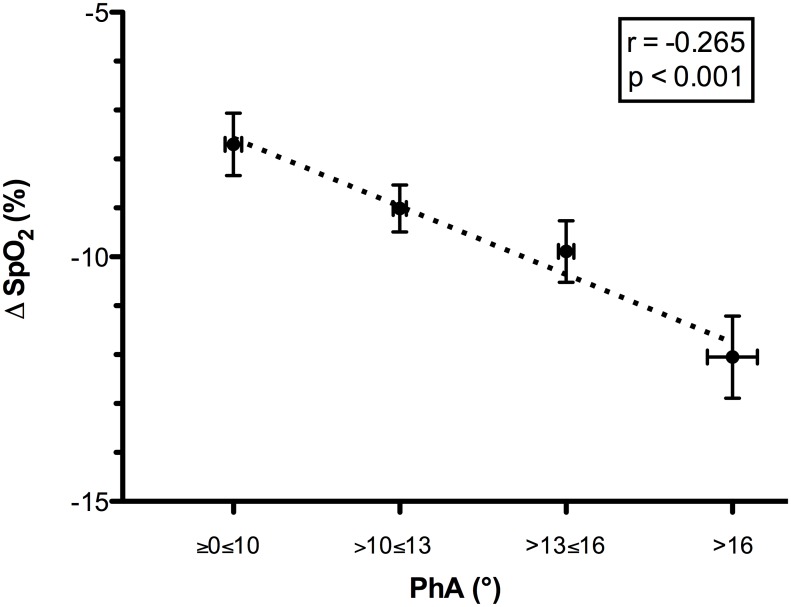
Relationship between the mean decrease in oxygen saturation and thoraco-abdominal coordination (PhA) during the run. The PhA values were grouped into four classes, each associated with the mean decrease in SpO_2_ (SpO_2_ at rest—mean SpO_2_ during the race). Reductions in thoraco-abdominal coordination, indicated by an increase in PhA, were significantly related to decreases in SpO_2_. Abbreviations: PhA, phase angle; Δ SpO_2_, mean decrease in oxygen saturation.

**Fig 3:**
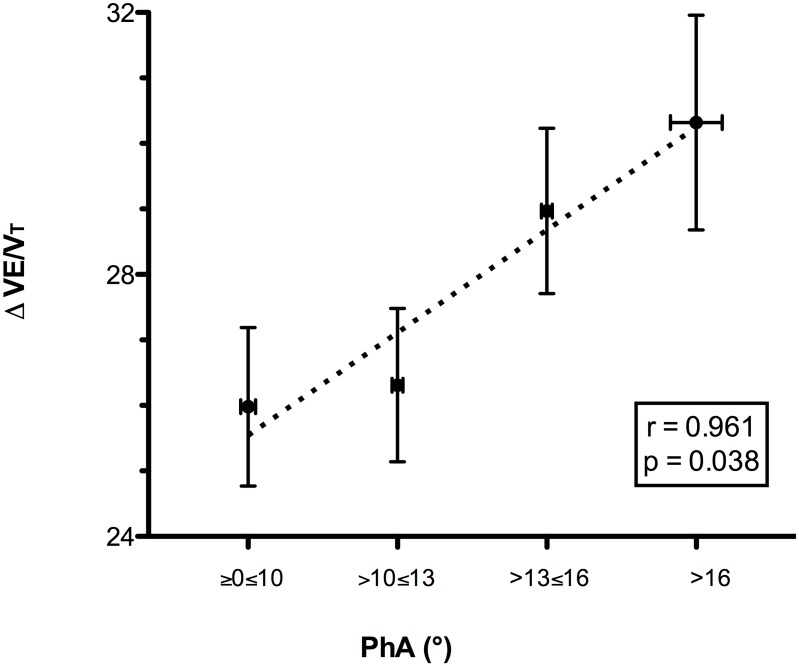
Relationship between the delta VE/VT ratio and thoraco-abdominal coordination (PhA) during the run. The PhA values were grouped into four classes, each associated with the delta VE/VT (difference between the VE/VT ratio during the run and at rest). Reductions in thoraco-abdominal coordination, indicated by an increase in PhA, were significantly related to increases in the VE/Vt ratio, which represents a less efficient ventilatory pattern. Abbreviations: PhA, phase angle; VE/VT, ratio between ventilation and tidal volume.

**Fig 4:**
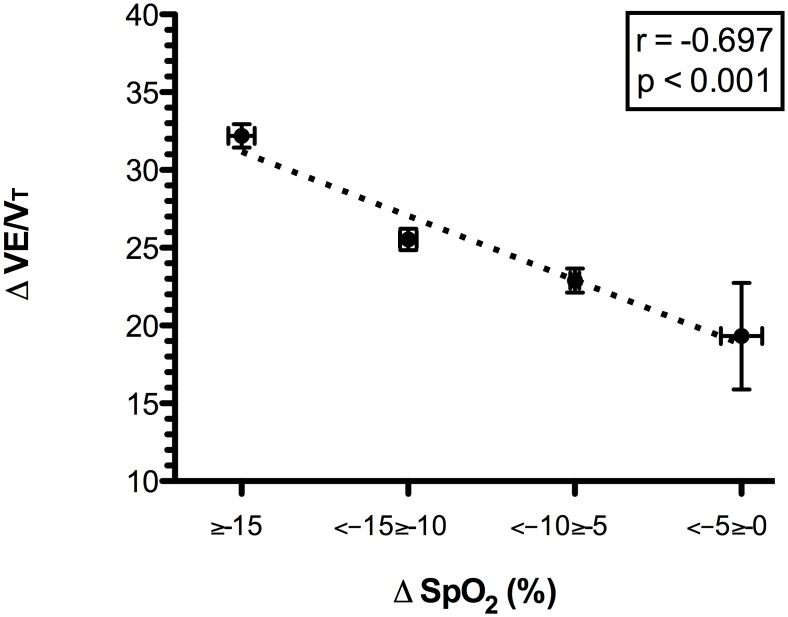
Relationship between the delta VE/VT ratio and the mean decrease in oxygen saturation during the run. Reductions in SpO_2_ (SpO_2_ at rest—mean SpO_2_ during the race) were grouped into four classes, each associated with the delta VE/VT (difference between VE/VT ratio during the run and at rest). Increases in the VE/VT ratio were related to greater levels of oxygen desaturation. Abbreviations: Δ SpO_2_, mean decrease in oxygen saturation; VE/VT, ratio between ventilation and tidal volume.

When the slope increased to over 30%, we observed a significant increase in PhA, indicating a decreased thoraco-abdominal coordination (Fig 1). The best interpolation of these points was represented by the following quadratic curve: PhA = 12.897–0.773slope + 0.301slope^2^ (r = 0.995, r^2^ = 0.990, F = 97.389, p = 0.036). Figs 2 and 3 present the mean decreases in SpO_2_ (calculated as SpO_2_ at rest—SpO_2_ during exercise) and increases in the VE/VT ratio (delta VE/VT), respectively, for each class of PhA. Increases in PhA were significantly related to decreases in SpO_2_ (r = -0.265, p<0.001; Fig 2) and increases in the VE/VT ratio (r = 0.961, p = 0.038; Fig 3). The increase in the VE/VT ratio significantly affected SpO_2_: greater decreases in SpO_2_ were associated with a higher VE/VT ratio (r = -0.697, p<0.001; Fig 4).

**Fig 5:**
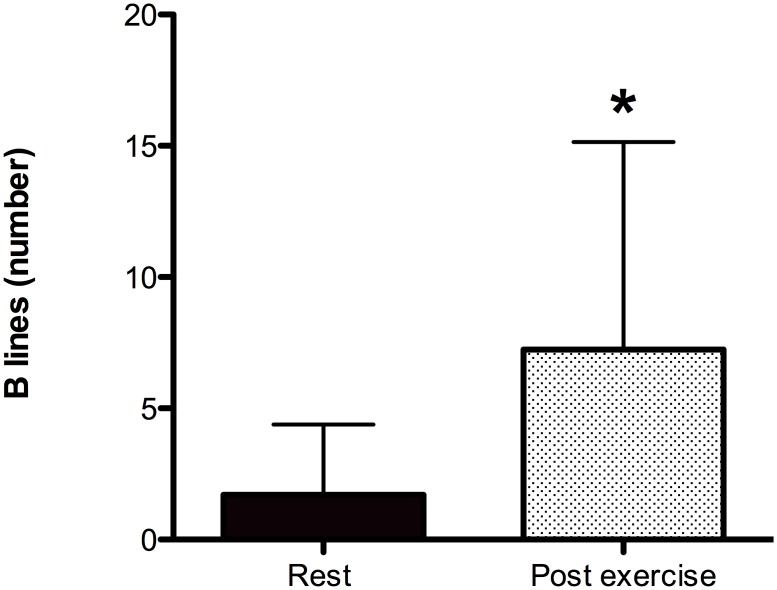
Thoracic ultrasound evaluation (US-B lines) at rest and after the run. Ultrasound echo evaluations performed before and immediately after the run showed increased interstitial lung edema, which was identified by the presence of US-B lines. *Student’s t-test, p<0.001.

The number of US-B lines, assessed via chest ultrasound examination, increased in 86% of the athletes after the run (US-B lines at rest, 2.0±4.2; US-B lines immediately after exercise, 7.5±7.2; p = 0.001); (Fig 5). The differences in the number of US-B lines were linearly correlated with the decreases in the SpO_2_ (r = 0.661, p = 0.032).

**Fig 6:**
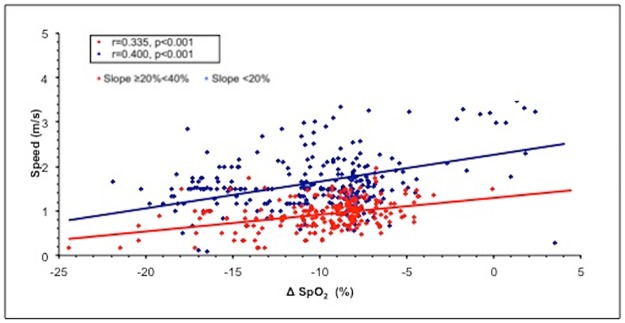
Relationship between mean horizontal speed and mean decreases in oxygen saturation during the run. To simplify the analysis, the slopes were grouped into two ranges: <20% and ≥20%. Abbreviation: SpO_2_, oxygen saturation.

Lower SpO_2_ values were associated with lower speeds at 20%≥slope≤40% (r = 0.335, p<0.001 for horizontal and r = 0.36, p<0.001 for vertical) and 0%>slope<20% (r = 0.400, p<0.001 for horizontal and r = 0.15, p = 0.01 for vertical); (Fig 6).

In the multivariable analysis, the mean decrease in oxygen saturation, the PhA and the slope remained independent predictors of both horizontal (β = 0.657, p<0.001; full model R adjusted = 0.207) and vertical speed (β = 0.543, p<0.001; full model R adjusted = 0.291).

Because the range of ages in the population was rather large (34–60 years), we tested for possible effects of age on both oxygen desaturation and speed and did not find a significant correlation (p = 0.924 and 0.092, respectively).

## Discussion

Studies on uphill running and mountain ultramarathons have considered biomechanical and neuromuscular adaptations [41,42] as well as respiratory modifications [3,7]. To the best of our knowledge, no study has investigated ventilation and ventilatory pattern during the run.

We hypothesized that during uphill exercise, the ground slope would influence the thoraco-abdominal coordination in breathing, which would modify the ventilatory pattern, possibly affecting gas exchange (i.e., SpO_2_) and athletic performance. Furthermore, we aimed to verify the development of asymptomatic interstitial lung edema after exercise that was strenuous but shorter than that described in the literature [18,19]. We studied a group of well-trained skyrunners during a run at moderate altitudes between 2000 m and 2800 m, where not only exertion but also mild hypoxia could affect the responses of the respiratory and cardiac systems.

### Ventilation monitoring

The main finding of our investigation is that ground slope negatively influences thoraco-abdominal coordination during intense exercise at moderate altitude, as shown by the increase in the angle representing the excursions of the two compartments. This phenomenon was more evident at slopes >30%, supporting reduced synchrony between the rib cage and the abdomen with increasing slope that we found in a previous study in which participants exercised at sea level on a treadmill at slopes between 0% and 25% [43]. We believe that the reduction in thoraco-abdominal coordination that resulted from increased slope was dependent on changes in posture, leading to a greater forward inclination of the trunk and a greater displacement of the center of mass to compensate for the increasing slope. Actually, a few studies on thoraco-abdominal kinematics [16,44] have reported an association between greater inclinations of the trunk and reductions in rib cage displacement as a cause of reduced coordination between these two compartments. Moreover, the reduction in thoraco-abdominal coordination in our subjects corresponded to changes in ventilatory patterns represented by an increased respiratory rate for the same amount of ventilation (i.e., a higher VE/VT ratio). This ventilatory pattern is less efficient in terms of gas exchange [10,13]. In fact, a ventilatory pattern characterized by a low respiratory rate and a high tidal volume is associated with a better maintenance of SpO_2_ and is therefore considered more efficient due to the reduction in dead space ventilation [10,11,13]. This phenomenon has been reported both in healthy subjects during exposure to high altitude and in patients with chronic respiratory and cardiac diseases [10–15,17].

In our study, changes in ventilatory patterns leading to decreased efficiency (i.e., higher VE/VT ratio) were associated with more severe decreases in SpO_2_, and this relationship was stronger for slopes >20%. As the slope of the terrain varied throughout the run [26] and was not correlated with the altitude, the reduced SpO_2_ observed during exercise at steeper slopes was not related to increased environmental hypoxia. Greater decreases in SpO_2_ appear to negatively influence performance: athletes with higher levels of oxygen desaturation during the run had slower speeds (both horizontal and vertical). In fact, horizontal and vertical speeds are considered the best indicators for endurance performance in mountain environments. The relationship between decreases in SpO_2_ and speed during this run at 2000–2800 m supports the role of arterial oxygen desaturation in the decline in performance at altitude, as reported by Chapman et al. [45] in a study of highly trained distance runners under conditions of simulated hypoxia (2100 m). Many factors can influence run speed, and our results show that a more severe reduction in SpO_2_ can contribute to affecting performance in highly trained subjects during heavy exercise in a mildly hypoxic environment.

### Respiratory function

Regarding lung function and respiratory muscle strength, our results are in agreement with data reported in the literature. Since the 1980s, it has been well known that athletes show a reduction in vital capacity and an increase in respiratory muscle fatigue after exercises of various intensities and durations [46–48]. In particular, during a 2.5-hour treadmill run, simulating a marathon, a reduction in vital capacity of approximately 3% was reported after at least 90 min, while an average 8.6% reduction in post-race vital capacity was found after a marathon [46]. A similar reduction of approximately 10% was recently reported after an ultramarathon [3,7, 49]. In our study we observed a small but significant 4% reduction in vital capacity after an uphill and downhill run with an average duration of 95 min. A possible cause of the decrease in vital capacity can be the reduced strength of the respiratory muscles, as reported by Vernillo et al [7]. A reduction in PImax is a sign of respiratory muscle fatigue, which can also play a significant role in performance limitations, contributing to reduced running speeds in prolonged running events [7, 50–52]. Accordingly, we found a trivial but significant reduction in PImax after the entire run. We must emphasize that our results were obtained after the downhill part of the run, in contrast to previous studies of similar duration, where the same results were obtained after a run on level ground [46, 47]. We wondered whether the descent could have affected the results. It has been reported that the energy cost of running is affected by slope in that cost linearly increases with increasing slope and linearly decreases with decreasing slope, with a minimum value at a -20% grade, after which it increases [41]. Therefore, even if the uphill run is more demanding in terms of cardiorespiratory work than the downhill run [41], the steepest parts of the descent can contribute to the cardiorespiratory work. In the trail covered in the present study, the steepest slopes were spread over the whole route and we think that the descent effort could have contributed to the results.

### Ultrasound lung echo

The development of interstitial pulmonary edema, as shown by the higher number of US-B-lines in the athletes after the run, was found in 86% of the subjects and was significantly related to reductions in SpO_2_. Similar increases in extravascular lung water have previously been described in outdoor environments in athletes immediately after both intense and prolonged exercise [18, 20] and in trekkers during high-altitude exposure with and without exercise [19, 53].

The pathophysiological mechanism of the development of asymptomatic interstitial pulmonary edema after intense exercise [18, 54] is related to different factors, such as increases in pulmonary blood flow [55] and the cardiac changes that occur during exercise [18].

We speculate that the changes in thoraco-abdominal coordination and the consequent decrease in breathing efficiency, which affects SpO_2_, can contribute to the development of asymptomatic interstitial pulmonary edema during intense exercise at moderate altitude. In the present study, the lung echo assessment was performed immediately after the descent, but we do not think that this timing affected the results. In fact, it has been reported that the radiographic evidence of mild interstitial edema persists for at least one and a half hours post-marathon [20]. Also the development of asymptomatic interstitial lung edema can be involved in the decrease in lung volumes, as previously reported in athletes after an ultramarathon [20].

To the best of our knowledge, this study is the first to analyze the combination of breathing coordination, ventilatory pattern, and SpO_2_ during exercise in the field involving continuous changes in ground slope. All these parameters were analyzed in relation to exercise performance and to the possible development of interstitial pulmonary edema.

We recognize that this study has certain limitations. One limitation is the absence of data regarding oxygen consumption and the work of respiratory muscles during the run to better identify their role in the performance. The lack of postural and kinematic investigation to support our suppositions constitute additional limitations. Finally, we did not control for the effect of increasing altitude alone (i.e., without exercise). We highlight that according to the literature and our own data [56, 25], the reduction in SpO_2_ between 2000 and 2800 m in healthy subjects at rest is approximately 4%. Therefore, at 2800 m, the SpO_2_ at rest is expected to be approximately 90%, a value higher than the values found in our study during heavy exercise.

## Conclusions

This study presents new data on ventilation during strenuous uphill exercise documenting the role that the respiratory system can play in performance under such conditions.

No study has yet monitored the ventilatory pattern and thoraco-abdominal coordination during an uphill run. The combined use of inductive plethysmography, which has been shown to accurately estimate ventilation during exercise [30], and GPS data provided us the unique opportunity to investigate ventilatory changes during this type of race.

In athletes performing intense exercise between 2000 m and 2800 m, steep slopes led to a reduction in thoraco-abdominal coordination, which was associated with a less efficient ventilatory pattern and decreased SpO_2_. These effects were more evident at slopes greater than 20–30%. Therefore, we suggest that ventilatory patterns play a crucial role in breathing efficiency and SpO_2_ and can be a contributing factor to declines in running speed and the development of interstitial pulmonary edema. Furthermore, we confirm the previous findings regarding the reduced strength of respiratory muscles and decreased lung function as well the development of interstitial lung edema after strenuous exercise.

From a practical perspective, these results reinforce findings indicating that athletes and trainers should pay particular attention to the respiratory system. Future investigations of posture during exercise at different slopes, at sea level, and under conditions of simulated hypoxia should provide additional information on the role of posture in determining ventilatory patterns.

## Supporting information

S1 Dataset Anthropometric, inductive plethysmography, SpO_2_ and GPS data. Abbreviations: BMI, body mass index; VT, tidal volume; VE/VT, ratio between ventilation and tidal volume; Ti, inspiratory time; Te, expiratory time; Tt total breath time; Ti/Tt fractional inspiratory time; Ti/Te, ratio between inspiratory and expiratory time; PhAng, phase angle; HR, heart rate; SpO_2_, oxygen saturation; delta SpO_2_, mean decrease in oxygen saturation.
(XLSX)
